# Correction: Three-dimensional-printed custom guides for bipolar coxofemoral osteochondral allograft in dogs

**DOI:** 10.1371/journal.pone.0259923

**Published:** 2021-11-09

**Authors:** Christina C. De Armond, Stanley E. Kim, Daniel D. Lewis, Adam H. Biedrzycki, Scott A. Banks, James L. Cook, Justin D. Keister

The fourth author’s name is spelled incorrectly. The correct name is: Adam H. Biedrzycki. The correct citation is: De Armond CC, Kim SE, Lewis DD, Biedrzycki AH, Banks SA, Cook JL, et al. (2021) Three-dimensional-printed custom guides for bipolar coxofemoral osteochondral allograft in dogs. PLoS ONE 16(2): e0244208. https://doi.org/10.1371/journal.pone.0244208
